# Selective Immunoglobulin M Deficiency in Patients with Autoimmune Liver Diseases

**DOI:** 10.5152/tjg.2024.23413

**Published:** 2024-06-01

**Authors:** Yasemin H. Balaban, Mohamed Ismail, Şefika Nur Ayar

**Affiliations:** 1 Division of Gastroenterology, Department of Internal Medicine, Hacettepe University Medical Faculty, Ankara, Türkiye; 2 Department of Internal Medicine, Rutgers New Jersey Medical School University Hospital, New Jersey, USA; 3 Department of Internal Medicine, Hacettepe University Medical Faculty, Ankara, Türkiye

The liver is well known for its metabolic and detoxification functions, but it also plays a crucial role in immunity. The liver can differentiate between harmless and harmful substances, although how this process is regulated needs to be delineated.^1^ Hepatic inflammation and dietary or microbial antigens may trigger an autoimmune process, and local failure to regulate the immune response can lead to autoimmune liver diseases (AILDs).^1^ Furthermore, AILDs were reported to be associated with inborn errors of immunity (IEI). Indeed, IEI can present not only with frequent or atypical infections but also with autoimmune diseases, including AILD, mainly autoimmune hepatitis (AIH). Besides being prone to AILD, patients with IEI also encounter challenges in terms of diagnosis and treatment.

Selective immunoglobulin (Ig) M deficiency (SIGMD) was defined as having a serum immunoglobulin IgM level that is repeatedly below 2 standard deviations of normal while maintaining normal levels of serum IgA, IgG, and IgG subclasses without associated T-cell defects or external factors that can be linked to the condition.^2^ Since the literature is limited on AILD associated with SIGMD, here we present the clinical features and prognosis of our 4 patients.

The database from Hacettepe University, Faculty of Medicine Hospital, was retrospectively reviewed for patients with AILD who were on follow-up at the adult gastroenterology outpatient unit between January and July 2020. These patients were retrospectively evaluated for the presence of IEI based on laboratory findings at the time of their initial diagnosis, prior to immunosuppressive therapy for AILD. Out of 82 patients with AILD, 4 patients had a diagnosis of SIGMD (unpublished data). Autoimmune hepatitis was diagnosed according to “The International Autoimmune Hepatitis Group” scoring system.^3^ Primary sclerosing cholangitis (PSC) was considered in patients with ALP elevation and cholangiographic evidence of bile duct changes after eliminating other causes for sclerosing cholangitis.^4^ Selective IgM deficiency was diagnosed based on the criteria set by the European Society for Immunodeficiencies.^2^

There are only 2 documented cases of AIH associated with SIGMD in the literature. Sano et al^5^ describe a case of a 21-year-old male in Japan, and Arahata et al^6^ describe a case of a 64-year-old female. In our case series, SIGMD was associated with AIH in 3 patients (a 33-year-old male, a 57-year-old female, and a 60-year-old female) and with PSC in 1 patient (a 36-year-old male). Per definition, patients with SGIMD exhibit low IgM levels and normal IgG levels. The IgM levels of our patients were higher (ranging from 40 to 87 mg/dL) in comparison to previously reported patients (2 and 11 mg/dL) (Table 1). Although patients with AIH typically have elevated IgG levels, Sano et al. suggested that the normal IgG levels (1,095 mg/dL) in their patient could be attributed to immune anergy resulting from IgM deficiency. On the other hand, Arahata et al^6^ proposed that the high IgG level (2942 mg/dL) in their patient was linked to the presence of cirrhosis. In our patients, IgG levels were normal, except for patient 4, who had a high IgG level (2700 mg/dL) in the absence of cirrhosis. Sano et al^5^ and Arahata et al,^6^ respectively, reported that their patients had negative autoantibodies and positive ANA at 1/160 titer. Two of our AIH patients had positive autoantibodies; LKM1 at 1/100 and ANA at 1/640 titer. Sano et al^5^ reported their patient presented with acute AIH and had remission after 160 days of steroid treatment. However, Arahata et al^6^ lost their patient because she refused steroid therapy and developed decompensated cirrhosis and HCC. All of our patients were non-cirrhotic and had a good response to AILD treatment, except patient 4, in whom azathioprine was switched to mycophenolate mofetil due to lymphopenia. Since our patients exhibited slightly reduced IgM levels and they did not have a history of frequent infections, intravenous immunoglobulin treatment for IEI was not administered to them.

Selective IgM deficiency was identified around 50 years ago, but it was not given much attention as primary immunodeficiency since patients with SIGMD may not show any symptoms, although they are more likely to have infections as compared to healthy individuals.^7^ While it is unclear how SIGMD leads to AILD, there are several proposed mechanisms. Immunoglobulin M acts as an early defense against microbes and plays a crucial role in maintaining an immune balance between protective inflammation and immune destruction or an autoimmune response. The ability of natural IgM to bind to self-antigens is essential for its protective function against autoimmunity.^8-9^ Immunoglobulin M antibodies react with conserved epitopes that are shared by both microbes and self-antigens. The production of IgM is triggered by interaction with self-antigens and the cross-linking of IgM Fc receptor (FcμR) and B-cell receptor, leading to the induction of anergy. Experimental studies using mouse mutants lacking FcμR demonstrated that a deficiency in IgM causes the targeted response of IgG antibodies against T-dependent and T-independent antigens, ultimately leading to the emergence of autoimmune conditions.^7^ The accumulation of CD21low B cells expressing high activation markers may also play a role in the initiation of an autoimmune response in patients with SIGMD. Additionally, it was shown that patients with SIGMD have a decline in CXCR3 expression on both naïve and memory B cells.^10^ The involvement of CXCR3 in T cells and T-cell-driven autoimmunity has been extensively documented, and its contribution to B cell migration and antibody-mediated autoimmunity is gradually becoming apparent. More research is needed to fully comprehend the role of decreased CXCR3 expression in SIGMD patients for the development of autoimmune conditions, including AILD.

This case series highlights the coexistence of SIGMD and AILD. The review of patients in the literature and this case series suggested that the underlying IgM deficiency might trigger AILD with a diverse range of clinical presentations and prognosis. The major challenges for the diagnosis of AILD in patients with SIGMD are the late age-onset of AILD, normal or decreased immunoglobulin levels, and negativity in autoantibodies. Therefore, the association of these 2 rare conditions must be considered in all types of AILD, especially those with atypical presentations or poor responses to treatment. Further studies are warranted to investigate the impact of SIGMD on the prognosis of AILD.

## Figures and Tables

**Table 1.** Clinical and Laboratory Features of Autoimmune Liver Diseases Patients with Selective Immunoglobulin M Deficiency

Variable	Patient 1	Patient 2	Patient 3	Patient 4
Gender	M	F	M	F
Age, years-old	36	57	33	60
Age at diagnosis, years-old	32	44	32	56
AILD	PSC	AIH	AIH	AIH
Associated autoimmune disease	Absent	Absent	Vitiligo	SLE, Sjögren syndrome, autoimmune thyroiditis
Organomegaly	Splenomegaly	Hepatomegaly	Absent	Hepatomegaly
Treatment for AILD	UDCA	Steroid	Steroid and UDCA	Mycophenolate mofetil*
Response to treatment	Good	Good	Good	Inadequate
Hemoglobin (M: 13.8-17.2 g/dL; F: 12.1- 15.1 g/dL)				
At diagnosis	15.8	14.7	16.7	14.0
At last visit	17.0	14.7	17.8	12.6
Leukocytes (4.0-10.0 × 10^3^/mL)				
At diagnosis	5.3	7.8	10.8	4.3
At last visit	5.5	7.9	7.5	3.2
Neutrophils (1.5-8.0 × 10^3^/mL)				
At diagnosis	2.7	4.6	7.2	2.2
At last visit	1.9	4.1	5.3	2.1
Lymphocytes (1.0-4.0 × 10^3^/mL)				
At diagnosis	2.1	2.7	2.6	1.7
At last visit	2.6	2.9	1.6	0.9
Thrombocytes (150-450 ×10^3^/mL)				
At diagnosis	247	286	228	154
At last visit	226	321	195	144
ALT (4-36 IU/L)				
At diagnosis	417	111	409	117
At last visit	34	42	22	72
AST (0-35 IU/L)				
At diagnosis	219	49	152	91
At last visit	25	25	21	55
GGT (8-38 IU/L)				
At diagnosis	111	18	325	108
At last visit	15	16	103	38
ALP (44-147 IU/L)				
At diagnosis	252	81	76	75
At last visit	145	73	40	78
Albumin (3.5-5.5 g/dL)				
At diagnosis	4.7	4.3	4.6	4.7
At last visit	4.9	4.4	4.9	5.0
Total bilirubin (0.1-1.2 g/dL)				
At diagnosis	2.4	0.8	0.8	0.9
At last visit	0.8	0.8	1.1	0.6
Autoantibodies ^**^				
At diagnosis	Negative	Negative	LKM 1/100	ANA 1/640
At last visit	ANA 1/100	Negative	ANA 1/100	ANA 1/320
IgG (913-1884 g/dL)				
At diagnosis	1550	1010	2760	1170
At last visit	1260	1040	1880	1720
IgA (139-378 g/dL)				
At diagnosis	Unknown	243	240	267
At last visit	158	209	229	198
IgM (88-322 g/dL)				
At diagnosis	77	44	74	76
At last visit	87	40	48	45
Lymphocyte subtypes				
CD3 (62%-88%) CD4 (35.3%-61.1%) CD8 (11.2%-37.3%) CD16/56 (3.2%-23.7%) CD19 (1.0%-3.6%)	69 44 23 22 6	75 53 27 16 5	66 45 20 19 4	90 53 41 2 4

AIH, autoimmune hepatitis; AILD, autoimmune liver diseases; ALP, alkaline phosphatase; ALT, alanine aminotransferase; ANA, anti-nuclear antibody; AMA, anti-mitochondrial antibody; ASMA, anti-smooth muscle antibody; AST, aspartate aminotransferase; F, female; GGT, gamma-glutamyl transferase; LKM, liver kidney microsomal antibody; M, male; pANCA, peripheral anti-neutrophil cytoplasmic antibody; PSC, primary sclerosing cholangitis; UDCA, ursodeoxycholic acid.

*Azathioprine was switched to mycophenolate mofetil due to lymphopenia.

**ANA, ASMA, LKM, AMA and pANCA were tested.
